# Community perception regarding childhood vaccinations and its implications for effectiveness: a qualitative study in rural Burkina Faso

**DOI:** 10.1186/s12889-018-5244-9

**Published:** 2018-03-06

**Authors:** M. Kagoné, M. Yé, E. Nébié, A. Sié, O. Müller, C. Beiersmann

**Affiliations:** 1 Centre de Recherche en Santé de Nouna, Ministry of Health, PO Box 02, Nouna, Burkina Faso; 2 Institute of Public Health, Medical School, Ruprecht-Karls-University, 69120 Heidelberg, Germany

**Keywords:** Community perception, Childhood vaccination, Qualitative study, Burkina Faso

## Abstract

**Background:**

Vaccination has contributed to major reductions in global morbidity and mortality, but there remain significant coverage gaps. Better knowledge on the interplay between population and health systems regarding provision of vaccination information and regarding health staff organization during the immunization sessions appears to be important for improvements of vaccination effectiveness.

**Methods:**

The study was conducted in the Nouna Health and Demographic Surveillance System (HDSS) area, rural Burkina Faso, from March to April 2014. We employed a combination of in-depth interviews (*n* = 29) and focus group discussions (*n* = 4) including children’s mothers, health workers, godmothers, community health workers and traditional healers. A thematic analysis was performed. All material was transcribed, translated and analyzed using the software ATLAS.ti4.2.

**Results:**

There was better social mobilization in the rural areas as compared to the urban area. Most mothers know the Expanded Program of Immunization (EPI) target diseases, and the importance to immunize their children. However, the great majority of informants reported that mothers don’t know the vaccination schedule. There is awareness that some children are incompletely vaccinated. Mentioned reasons for that were migration, mothers being busy with their work, the practice of not opening vaccine vials unless a critical number of children are present, poor interaction between women and health workers during immunization sessions, potential adverse events associated with vaccination, geographic inaccessibility during rainy season, and lack of information.

**Conclusions:**

Well organized vaccination programs are a key factor to improve child health and there is a clear need to consider community perceptions on program performance. In Burkina Faso, a number of factors have been identified which need attention by the EPI managers for further improvement of program effectiveness.

## Background

In 1974 the World Health Organization (WHO) started the Expanded Program on Immunization (EPI) with the aim to vaccinate children all over the world [1]. Standardized vaccination schedules have been developed and are regularly updated. The EPI has been shown to achieve and sustain high levels of immunization coverage in many but not all countries which ensures effective protection of children against several infections [2]. Moreover, the EPI has contributed significantly to global reductions in childhood morbidity and mortality [3, 4]. Substantial efforts have been made by sub-Saharan African (SSA) countries to reinforce their immunization programs [5]. However, coverage and quality of vaccination efforts remain important challenges. WHO and the United Nations Children’s Fund (UNICEF) estimate that 1.5 million children worldwide continue to die from vaccine-preventable diseases every year because of inadequate vaccination coverage mainly in SSA and South-East Asia [6, 7].

Several factors can explain why children are not completely vaccinated. Immunization can be inadequate due to delayed vaccination (i.e. vaccinations are not provided in time), incomplete vaccination (i.e. some vaccinations are missing) or no vaccination (i.e. the child did not receive any vaccine). To receive age-appropriate doses of antigens, children need to be taken to the health facility by their parents. To avoid vaccine wastage and to reduce costs, vaccines are supplied in multi-dose vials. Many countries have restrictive policies for opening these vials. Such restrictive vial-opening policies are a known factor for delayed vaccinations [8, 9].

Apart from operational factors relating to policies and economic considerations, vaccine availability and health worker related factors, as well as awareness, attitude and perception of parents have been identified as major obstacles to high immunization coverage [10, 11]. When programs with the goal to increase immunization coverage are developed and implemented, knowledge, awareness, attitudes, beliefs and circumstances of the concerned populations are unfortunately often ignored and not well documented [12]. Special emphasis should thus be placed on listening to concerns and understanding perceptions of the community and parents regarding reasons why some children are incompletely vaccinated or not vaccinated at all [13]. This has become even more important with the increasing awareness on the importance of non-specific effects of childhood vaccinations [14–19].

For Burkina Faso, only a few vaccination studies touched upon qualitative aspects such as e.g. vaccination perceptions of the population or specific population groups [20–24] Against this background, the aim of this study is to determine the reasons related to complete and incomplete immunization coverage among children in a region of rural Western Burkina Faso, with a focus on the communities’ perception and the provision of immunization services. Specific study objectives are to understand perceptions of mothers and health workers regarding the vaccination program (1), to identify factors that influence immunization coverage (2) and to make recommendations on how to improve the vaccination program (3).

## Methods

### Study area

The study was implemented in collaboration with the Centre de Recherche en Santé de Nouna (CRSN) which is located in the Nouna Health District (NHD), 300 km from the capital Ouagadougou in north-western Burkina Faso. Since 1992 a Health and Demographic Surveillance System (HDSS) is established in the research zone of the CRSN. It covers an area of 1775 km^2^ and consists of 58 villages and Nouna town with some 90.000 inhabitants living mostly in the rural area [25]. The study area is a Sub-Sahelian dry orchard savannah with a long dry season (November–May) and a short rainy season (June–October) [26].

The routine vaccination program in Burkina Faso recommends seven different vaccines for the prevention of eleven infections: (1) Bacillus Calmette-Guérin (BCG), (2) Oral Polio Vaccine (OPV), (3) Pentavalent Vaccine for diphtheria, tetanus, pertussis, hepatitis B, and Haemophilus influenzae type b (Penta), (4) pneumococcus vaccine (PCV), (5) rotavirus vaccine (ROTA), (6) yellow fever vaccine (YFV), and (7) measles vaccine (MV). The recommended vaccination schedule in Burkina Faso is BCG and first dose of OPV (OPV0) at birth, first dose of Penta (Penta1), OPV1, ROTA1 and PCV1 at 8 weeks, Penta2, OPV2, ROTA2 and PCV2 at12 weeks, Penta3, OPV3, ROTA3 and PCV3 at 16 weeks, and MV and YFV at 9 months of age. Peripheral health centers (CSPS) cover villages in the rural areas. Here, monthly vaccination sessions are organized to cover children’s routine vaccinations either in the village of the CSPS or through outreach visits to other villages. In Nouna town, there is the possibility to receive vaccinations daily in the urban CSPS [27].

The percentage of children completely vaccinated in the HDSS was 80.50% [28]. For the NHD the administrative coverage - based on the number of vaccine doses administered to an estimated target population [29] - was 106.8% in 2013 while the national coverage was 98.4% [30].

### Study design

This is a descriptive qualitative study which focused on communities’ knowledge and perceptions towards routine childhood vaccination. Semi-structured interviews, focus group discussions (FGD) and observations were the tools of data collection. This qualitative research allows researchers to better understand phenomena in context-specific settings [31]. It is thus a fruitful way to explore people’s needs, experiences, attitudes, thoughts and perceptions of different phenomena [32].

The study was conducted in NHD from March to April 2014.

### Study participants

Participants include immunization program managers who deliver vaccines to health facilities and compile vaccine reports, health workers responsible for performing vaccination, “godmothers” (“*doctoro mousso*”, community women leaders), community health workers (CHW) who disseminate information about immunization schedule in their community, traditional healers who provide traditional health care measures to children, and mothers of children less than three years. CHWs, godmothers, and traditional healers are part of a community as leaders, and take on multiple roles and responsibilities regarding any health problems in the community. They can represent the community for questions regarding population health and are fathers and mothers of children themselves.

The participants of the study were recruited in nine villages and Nouna town, representative of the five major ethnic groups residing in the HDSS area (Dafing, Bwaba, Mossi, Peulh and Samo).

### Data collection

Interviews were performed in French with EPI program managers and health workers and in the local language Dioula for other participants. Interviews were conducted by the first author of this paper (MK). A total of 29 interviews were conducted. Nine in-depth interviews were conducted with health workers in health facilities and two with program managers. At community level, six godmothers, seven traditional healers, two community health workers and three mothers were interviewed. These interviews focused on strategies used in rural and urban areas to improve immunization coverage and on the functioning of the vaccination program. They also helped to understand the perceptions and opinions of people on the organization of vaccination sessions and its usefulness,. Other factors highlighted included difficulties encountered during immunization sessions, recommended solutions, and further factors that influence vaccination uptake.

A total of four FGDs were performed; one FGD with mothers during a vaccination session, one FGD with godmothers and two FGDs with health workers. The number of participants in the FGDs was between five and nine. The conduction of the FGDs permitted to obtain impressions and views of the participants of the FGDs regarding the vaccination sessions: the perspectives of the views of the mothers as participants of the vaccination sessions, and the perspectives of the godmothers and health workers involved in the planning, organization, and operation of the sessions. The FGDs concentrated on identifying positive and negative situations encountered or experienced by mothers during the vaccination sessions as well as the behavior of health workers during the vaccination sessions. Moreover, aspects of health-worker-mother interactions during immunization sessions were captured during the FGDs as recognized by health workers and local disease prevention measures used by mothers. The FGDs focus not on individual views or expectations, but tried to identify the social representations, the common ground, and stimulate debate within those groups.

### Data analysis

The study focuses mostly on the manifest content presupposing that statements in the individual interviews and in the FGDs are complete units from which we identified emerging themes [33]. Classical thematic analysis was used [34]. Data were reduced by codification through assigning the same code to the same or similar unit of meaning [35]. Codes were generated moving through the data applying a mix of deductive and inductive coding. Principal themes and sub-themes were identified. Evidence that support or denies each theme and sub-theme and links between them were sought. The health belief model was used to develop the coding scheme. Based on this model, perceived susceptibility, perceived severity, and perceived benefits are likely to be positively related to immunization behavior [36].

The analysis was performed using the software ATLAS.ti4.2.

## Results

### The organization of the vaccination program in NHD

Provision of immunization services is one of the duties of health workers working in governmental health centers. They are responsible for the provision of services to populations in their center’s catchment areas. CHW and godmothers are representatives of the community who provide a link between the later and health workers. They are chosen by the community that they represent in the CSPS. When there are health problems, they are supposed to address such problems in collaboration with health workers. Their primary responsibilities regarding immunization are to provide information to mothers regarding EPI diseases, encourage women to take their children for vaccination, and inform about the immunization sessions. Each month, one task of the godmother is to educate parents, especially mothers, on various topics related to vaccination, including the importance of prevention for children and dates of immunization sessions.

*“Godmothers distributed the vaccination card. On vaccination days we sensitized when there are many women; for example when we have got about 20 women, we did the sensitization before vaccinating.” *(Informant 3, health worker)

*“What we are doing now is to keep the vaccination cards of children who have not yet received BCG in the CSPS and to inform them on this vaccination during the next session. And once the child is vaccinated, there is a number that was put on the vaccination card and the same number will remain on a list in the health facility; so we use this list in subsequent vaccinations to find those who are absent”* (Informant 2, health worker)

### The different vaccination strategies used in rural and urban areas to improve immunization coverage

In the rural area, many people are involved in preparing a vaccination session – CHW, village chief, griots (a poet, itinerant storyteller who passes the oral tradition and information in a community), and godmothers. There are some social pressures on mothers to bring their child the day before the immunization session to a specified place where the CHW gives the vaccination card to every mother. The CHW knows the village and the mothers. If one stays absent, the CHW will reach out and look for her. This procedure is like a convocation, and every mother feels obliged to bring her children. The outreach vaccination teams are functioning in the rural areas. This practice is different in the urban area where godmothers just give oral information to the parents. Mothers in the urban area have to seek vaccination in the health facilities.

*“Before vaccination in the community, CHWs are informed a day before, and in turn tell the mothers. Immunization cards are usually given to the mothers a day before.”* (Informant 3, health worker).

*“For example, now we are doing the program of April and three days before each session of vaccination, I inform the CHWs, I give them the individual vaccination cards of children and the day before the session, cards are distributed to each mother at home, and the day of the vaccination, they all come*.*”* (Informant 10, health worker).

In the rural areas everybody knows each other, which makes looking for absent mothers and children in the village easier. The way the vaccination sessions are organized in the rural areas puts a kind of social pressure on the women and the system of looking for the absent mother’s in the village hence leads to better coverage.

*I think in rural areas vaccination activities are easier and simpler because whatever the size of the village, as people live in community, we have all the children in the village. On the other hand in urban area in the city of Nouna it is complicated because there is not a mixing, you may not know your neighbor. Even if there are health workers per area they cannot control the children.* (Informant 22, *Program manager 1*).

*There is a system of children's search that each CSPS has put in place, it means from the vaccination register book, they know if the child must come today for his vaccination, the names of those who are absent are recorded and their names are given to the CHWs who assist the health workers in the vaccination.* (Informant 22, *Program manager 1*).

### Community knowledge and perceptions towards routine childhood vaccination

Regarding mother’s knowledge on the diseases targeted by the vaccination program, most informants (22/26) reported that women know the EPI target diseases. Diseases are explained in the local language. Young mothers are often those who don’t have full knowledge of these diseases. But it became clear that most people knew the common diseases that immunization could protect children from.

*“There is no problem; women have mastered the EPI target diseases because sensitization is done in local language with the community health worker. Most women have participated one or more times in EPI activities. Some women even serve as an educator relay for others to raise awareness on these antigens”* (Informant1 FGD, health worker).

*“Today, young mothers have no longer seen the devastation these diseases have; they are thus not motivated to come to vaccinate their children. For them, it is the pain they are afraid of; they don’t have fear of the disease itself”.* (Informant 6, health worker)

The EPI program diseases reported by the women are poliomyelitis, measles, yellow fever and meningitis.

*“The EPI target diseases are poliomyelitis, measles, and yellow fever.”*

*“There is also polio, and meningitis”*. (Informant 9, FGD mother).

Although most of the women knew the EPI target diseases well, many informants reported that mothers don’t know the EPI program itself, i.e. the schedule of the vaccinations, and their different appointments.

For every immunization session, the parents of children need to be called by health workers to participate. And not every woman participates.

*“At the vaccination level there is no problem, the problem is that more than half of the population is illiterate, so you have to remind them every time on their appointments so that they bring their children for immunization. ”* (Informant 10, health worker).

*“There are some who know, but others don’t know. Even those who know are still waiting for information. If you don’t go to inform them, they will not come, and they will say that it is not the time yet. It is a duty to us to tell them; otherwise, some will delay the immunization session”* (Informant 8, health worker).

“*They come to call us every day, I tell them that I am coming and I don’t go”* (Informant 5, mother).

Regarding the importance of childhood vaccination, most interviewees indicated that mothers of children know the importance of vaccines for their children. They explain it as the way of protecting a child from deadly childhood epidemics and a sure way of having a healthy child.

*“There is nothing better than vaccination. When your child gets all the vaccines he is protected from many childhood diseases. Today and through immunization, many illnesses disappear, and God helps also. All women today know the benefits of vaccination and also in the case of a little fever they send the child to the health facility. We suffered in the early days of vaccination when we must run after some women 4-5 times before they brought their children.”* (Informant 19, CHW)

*“Vaccination is good because through vaccination all diseases that existed when we were little are gone today - for example measles and smallpox which caused epidemics when we were small and in school”* (Informant 9, FGD mother).

In most cases, participants get information on immunization from their health workers at CSPS, from CHWs and godmothers, and through the radio. It was mentioned by health professionals and clients of vaccination services that before immunization, health education sessions are regularly conducted by health workers. Topics discussed during these health education sessions are for instance the importance of vaccinations, the diseases they protect against, vaccination procedures and their side effects.

Other sources of information on immunization included mosques and churches. People at the community level who play a significant role here regarding vaccinations include religious leaders, griots (traditional musicians and storytellers, societal leaders and guards of the oral tradition), and village administrative officers who pass on the message about immunization to mothers and other people in the community.

*“There was first the information we give to the community, we communicate the date of vaccination, and we choose the target children for vaccination. In our particular case, we send the children records form with the list of children to the CHW. In addition to that, the black cherry or town crier moves around before the vaccination, usually at night to provide information in a similar format. CHW, delegate and village advisor [*community administrative leaders*] work together to choose the place for the vaccination session and on that day we leave early for the vaccination work.”* (Informant 14, health worker).

### Reasons for incomplete or delayed vaccination

Mobility, business and concerns about the safety of vaccination were among the main reasons given by respondents to explain why children had delayed, or missed vaccinations.

*“Often there are some days we have no time because of the work so we decide to come the next day.”* (Informant 9, FGD mother).

*“This is related to frequent trips during events such as funerals, weddings, etc ... For example, today I missed five children in DAR SALAM to be vaccinated in the 9th month for reasons of marriage in Mali where they originate.”* (Informant 14, health worker).

*“There is too much mobility in civil servant households. They can start vaccination and from one day to the next she [*the woman*] or the husband is affected, and this poses a problem”* (Informant 4, health worker)*.*

*“I told you this is commerce, for example during the mangoes season, women go in the early morning to sell their products. We inform them, but they refuse to stop their business for that time and bring their children for vaccination.”* (Informant 18, CHW)

*“In our villages, some women do not always see the benefits of vaccination, even if they are not numerous, they still exist. Women who are not sufficiently aware of the safety of vaccination, they often flee if their children after vaccination running a fever for 48 hours.”* (Informant 22, health worker).

Other determinants are in the sociocultural area. When the woman gives birth, in some ethnic groups and religions, the child should stay at home until the name giving ceremony. In this situation there are children who missed or delayed the first vaccines.

*“The problem for Moslems here in Koro is that the woman must stay in the house after childbirth until the 8th day; often we even have the problem for our postnatal [*visit*], we give a 7-day appointment to the woman and she comes back on the 10th day.”* (Informant 1, health worker).

Another practice is the move of the woman to give birth in the parent’s village.

*“Some women went to their village of origin, at their parents’ place, to give birth, and therefore if the CHW could not detect these women at time, it is sure that their children won't receive the vaccines.”* (Informant 1, health worker)

Reaching the number of children required for opening a vial of vaccines is one reason to delay vaccination among children. For example, BCG is supplied in vials with 20 doses and measles vaccine in vials for 10 doses; and once opened the vaccine should only be used for a maximum of 6–8 h. To minimize the loss of doses, the health system adopted the policy of not opening a BCG vial unless 10 children are present to be vaccinated and 5 to 6 children are required for opening a measles or a Yellow Fever vaccine vial.

*“We are always explaining that there is a dose for 20 children so if we open it and if we can’t find the 20 children the remaining product will be spoiled because we can’t keep it. …For yellow fever and measles, if the number of children is not reached, vaccine books are removed and we give an appointment [*to the mothers*] for the day of vaccination in a health facility.”* (Informant 1, health worker)

At the beginning of the immunization session, health workers explain to mothers why the vial of vaccination should not be opened if the expected number of children is not reached.

*“Often we think they are right, they are told to come early in the morning, and she comes and since children who have to take BCG are not many she must wait while we vaccinate those concerned with the PENTA; this is problematic, the waiting time is often long. … It's always quiet and explains that there is a dose for 20 children so if you open it and you don’t find the 20 children, the remaining product will be spoiled because you can’t keep it. They get irritated about, but they accept the principle.”* (Informant 1, health worker)

Poor interaction between women and health workers during immunization sessions is also one factor which explains incomplete vaccinations among children. Mothers mentioned a bad welcome while arriving at the vaccination session, particularly for those who lost their child’s vaccine card. This is then an argument to delay vaccination. In addition, long waiting time was also criticized. This situation is explained by the non-compliance with the order in which women arrived (related to reaching a sufficient number of children to open a vial).

*“The vaccination doesn't begin early, often you come early [*morning*] and you wait until 11 o’clock and the vaccination session doesn't start. Often also the order of arrival is not respected, there are some women they arrive after you and their children will receive their vaccines before you.”* (Informant 9, FGD mother).

“*For BCG, 20 children are necessary because we have small bottles of 20 doses. It is the same for measles; the vaccine is conditioned in small bottles of 10 doses. It makes some women wait for a longer time than others”* (Informant 15, health worker).

The health workers recognize that mothers often wait for a long time during the immunization session before their children receive for example a BCG vaccine.

*“Often, we give them [*the mothers of the children*] reason because we tell them to come very early in the morning and they arrive and as children who must receive BCG are not numerous, they must wait until we vaccinate those concerned by PENTA; all this situation poses problem; they are often waiting for long time”* (Informant 1, health worker).

Geographical inaccessibility during rainy season is also an issue. Some villages are far from the health facility and inaccessible during the rainy season.

*“It is not the whole population who has access to the health facility; during the rainy season the CSPS is inaccessible for many.”* (Informant 1, health worker)

Lack of information was also noted.

*“They don't know the day of vaccination; the sector is very vast, there are some women who don't receive information.” *(Informant 20, CHW).

Illiterate mothers lack knowledge on the benefits of vaccines. The decreasing number of epidemics and hence the diseases no longer being visible in the villages, makes people not see the need for vaccination. This is especially true for young mothers.

*“To be honest with you I don’t like vaccination. Unless I change one’s mind afterwards. I'm not saying it's not a good thing, but I do not like vaccination”* (*Informant 5, mother)*

*“Young mothers that we have nowadays didn't see the devastations that these illnesses [epidemic] made; they are not motivated to come to vaccinate their children.”* (Informant 1, FGD health worker).

*They don't understand the soundness of the vaccination; the moms themselves don't know why we vaccinate their children*. (Informant 2, CHW).

“I think it is laziness.”

“For others it is ignorance.”

“For those who don’t know the importance of these vaccines think they spend time for nothing.”

*“Others say that even if they do not come to the vaccination session there is no problem.”* (Informants, FGD mother).

Misinformation or rumors about vaccinations still exist in some villages and might affect the uptake of vaccinations.

*“They flatter themselves, some say these vaccines are not of quality, others say if they vaccinate your small boy with these vaccines, when he will get married he won't be able to make a lot of children.”* (Informant 8, FGD Godmothers).

*“Me I had the argument because of the vaccine against polio: there are some people who dismissed us from their home, saying that we put false products in the mouth of their children and also that we give some products to the women to spoil their pregnancy.”* (Informant 8, FGD Godmothers).

Some mothers recognize side effects after their children were vaccinated, e.g. fever, children crying over extended periods after vaccination or a swelling after being injected. Some respond to the side effects through giving the child painkillers while others go to the hospital for proper management. Side effects might be a reason for mothers dropping out of the vaccination program.

“Some are afraid of children crying after vaccination, so they no longer return to vaccination.”

*“They say, even if they don’t come, the child will not have a risk to get the illness”* (Informants, FGD mother).

*“When a woman comes home and the child is crying, and if she did not sleep, at the next vaccination she will be reluctant. They say it will be like last time. Otherwise, we give women all immunization-related information to ensure that they do not break the attendance of vaccination. But despite all this, there are some women who are still reluctant”* (Informant4, health worker)*.*

*“Last month, there's a new health worker who has vaccinated my child and afterwards his thigh was swollen.”* (Informant 9, FGD mother).

Health workers behavior during the vaccination session was mainly appreciated positively. But as far as technical knowledge is concerned, mothers felt that some health workers didn’t perform well. This situation was especially the case for trainees.

*“My child was vaccinated first by a man and when we come for the next vaccine, a woman gave the injection. I did not appreciate the way she had vaccinated my child. She caused a lot of harm to children, and she pushed the needle too deeply.”* (Informant 9, FGD mother)

*“It should be avoided to give children to the trainees who have not yet experience. Let them observe first how nurses do it before giving them children to be vaccinated”* (Informant 9, FGD mother).

## Discussion

### The organization of the vaccination program in NHD

CHWs have an important role in the implementation of immunization strategies. They are responsible for informing, sensitizing and mobilizing people. They go door to door asking women to meet the appointment set by the health worker. It can also happen that a “griot” ensures this role. The essential element that is recognized by all interviewees is that health professionals always go through CHW, godmothers or other community leaders to access children for vaccination. This is in line with another study in Burkina Faso [37]. CHWs are also known to have an important role in the organization of vaccination programs in neighboring countries such as Mali and Côte d’Ivoire [38, 39].

### Community knowledge and perceptions towards routine childhood vaccination

This study provides information of community perceptions regarding routine vaccination in rural Burkina Faso. In some countries and regions, studies revealed misinformation, rumors, or perceptions of vaccination that differ from the biomedical view [40]. For example, a study in Nigeria showed that mothers erroneously believed that their children would not suffer the diseases even if not immunized [40]. The current study provided information regarding perceptions of childhood vaccination among communities in a region of rural Western Burkina Faso. Here, routine vaccination is mostly well accepted by the mothers and the importance of child protection against deadly infectious diseases is known by all study participants. These findings support similar findings from low-income countries on this topic [27, 41, 42].

A vaccination coverage study in the same research area found better vaccination coverage in rural as compared to urban areas: full coverage in children aged 12–23 months was around 75% [41]. Another study arrived at the same conclusion that rural children have an advantage over urban kids [15]. This difference is likely attributable to the outreach vaccination teams functioning in the rural area (i.e. health workers actively reaching out to the rural population, making sure women attend the vaccination sessions) while mothers in the urban area have to seek vaccination in health facilities (i.e. the decision and effort to go to a health facility for vaccination is left to the women). The way the vaccination sessions are organized in the rural areas puts a kind of social pressure on the women: in the villages everybody is aware of who came to the vaccination session and who not. Although there is no “punishment” if a mother does not show up with her child for vaccination, the system of looking for the absent mothers in the village (visiting the homes of the absent mothers by the vaccination teams) hence leads to better coverage. In addition, the number of CSPSs increased in the rural area: Between 2009 and 2013, 233 new CSPSs opened in Burkina Faso, an increase from 1373 in 2009 to 1606 in 2013 [30].

Most women know the EPI target diseases. Diseases are explained in the local language during the vaccination session. However, those who don’t have full knowledge of these diseases are often young mothers. This supports former findings in Burkina Faso, The EPI targets diseases are well known and classified among the illnesses of the “white people” [43].

The informants reported that mothers often don’t know the program due to illiteracy. In the area of Nouna, about 65% of the population of 7 years and more have no education [14]. In this framework, knowledge of dates and places of immunization sessions are all crucial. Health workers have to remind women every time on their appointments so that they bring their children for immunization. Moreover, the possibility of side effects significantly influence the behavior of mothers, which supports similar observations in other populations [43–45]. Finally, mobility of respondents was also shown to be a serious barrier to vaccination, supporting similar observations from other studies [46].

### Reasons for incomplete or delayed vaccination

An important barrier for the uptake of child immunization identified in this study resides in the healthcare system: the policy of not opening a vaccine vial if not enough children are available. This barrier has received already considerable attention in the literature and thus needs more attention [37, 42, 47, 48].

Regarding health workers’ behavior during the vaccination session, this study finds that health workers are generally kind with mothers during the vaccination session. A study in Burkina Faso shows that the behavior of the health workers constitutes the determining component of vaccination coverage. He must create a climate of confidence with the population who will then accept to vaccinate their children, as far as the service is available [43].

In the majority of cases, mothers reported that vaccinators behaved well and as far as their technical knowledge is concerned, they felt that these professionals – with the exception of trainees - were performing well. Only occasionally participants said that vaccinators were rude and shouted at mothers, or they felt that vaccinators did not inject well and injured children during the process. That vaccinators do not provide vaccinations when there are only a few children present or if mothers come late causes discontent in the mothers. A study in Nigeria found a similar result [49, 50].

Health workers attitudes and behavior were mainly appreciated by the mothers, although some complained about the limited technical capacity of trainees without experience. Similar positive appreciation of health staff behavior has been reported from Uganda and the Dominican Republic [51].

Misinformation or rumors about vaccinations is common practice in West Africa. A study in Benin reveals e.g. a from the biomedical perspective erroneous perception of child vaccination. Those who are reticent say vaccination goes against the will of God, that it is a poison from the “white witch doctor” [52]. Another study in Nigeria shows that mothers erroneously believed that their children would not suffer the diseases even if not immunized [40].

### Strengths and limitations

The strength of the study is that the author himself has done the interviews in local languages and in French which will reduce translation bias.

A limitation of the study is that respondents were selected from only one district in Burkina Faso and may thus not be fully representative for the situation in this country.

### Recommendations

Restrictive vial-opening policies to avoid vaccine wastage are in place in many countries. Yet such restrictive policies are a known factor for delayed vaccinations. In this light we strongly recommend the development of low-cost, single dose vaccinations.

Recommendations to improve coverage start with reduction of missed opportunities by creating collaboration among health facilities during the vaccination sessions. This collaboration could also expand the vaccination area.

With a system of community health workers in place, sensitizing mothers and emphasizing messages about the benefits of vaccination sessions is important. A special focus should here be placed on the challenge of migration/movement of mothers without the vaccination card. Information and education of mothers about the importance of the card and the possibility and necessity of bringing the card to and getting vaccinations at health facilities elsewhere should be highlighted.

## Conclusions

The study has provided further evidence for the importance of knowledge on community perceptions regarding improvement of childhood vaccination program effectiveness. To achieve the national objective of complete and timely immunization coverage of 80% of eligible children, there is a need to consider the factors identified as barriers to the uptake of immunizations. In particular, there is a need to revise the policy of opening of vials only if a certain number of children is available. Such a change in policy will likely also increase the efficacy of the vaccination schedules due to the positive non-specific effects of childhood immunizations.

## References

[1] OMS. Le point sur les vaccins et la vaccination dans le monde OMS/UNICEF/Banque Mondiale. 2003. p. 1–96.

[2] UNICEF. Progrès pour les enfants: un bilan de la vaccination. UNICEF. 2005:1–17.

[3] Plotkin SA, Plotkin SL (2011). The development of vaccines: how the past led to the future. Nat Rev Microbiol.

[4] Andre FE, Booy R, Bock HL, Clemens J, Datta SK, John TJ (2008). Vaccination greatly reduces disease, disability, death and inequity worldwide. Bull World Health Organ.

[5] Bicaba A, Haddad S, Kabore M, Taminy E, Feletto M, Fournier P (2009). Monitoring the performance of the expanded program on immunization: the case of Burkina Faso. BMC international health and human rights..

[6] Black RE, Cousens S, Johnson HL, Lawn JE, Rudan I, Bassani DG (2010). Global, regional, and national causes of child mortality in 2008: a systematic analysis. Lancet.

[7] World Health Organization (2012). United nations children's fund(WHO/UNICEF). Global immunization data.

[8] Thysen SM, Byberg S, Pedersen M, Rodrigues A, Ravn H, Martins C (2014). BCG coverage and barriers to BCG vaccination in Guinea-Bissau: an observational study. BMC Public Health.

[9] Jani JV, De Schach C, Jani IV, Bjune G (2008). Risk factors for incomplete vaccination and missed opportunity for immunization in rural Mozambique. BMC Public Health.

[10] Feikin DR, Lezotte DC, Hamman RF, Salmon DA, Chen RT, Hoffman RE (2000). Individual and community risks of measles and pertussis associated with personal exemptions to immunization. JAMA.

[11] Sharma R, Bhasin SK. Routine immunization - do people know about it? A study among caretakers of children attending pulse polio immunization in East delhi. Indian journal of community medicine : official publication of Indian Association of Preventive & Social Medicine. 2008;33:31–34. 10.4103/0970-0218.39240 PMC2782225 19966993

[12] McCormick LK, Batholomew LK, Lewis MJ, Brown MW, Parental KH (1997). Perception of barriers to childhood immunization: results of focus groups conducted in an urban population. Health Educ Res.

[13] Larson HJ, Cooper LZ, Eskola J, Katz SL, Ratzan S (2011). Addressing the vaccine confidence gap. Lancet.

[14] Aaby P, Martins CL, Garly ML, Bale C, Andersen A, Rodrigues A (2010). Non-specific effects of standard measles vaccine at 4.5 and 9 months of age on childhood mortality: randomised controlled trial. BMJ.

[15] Shann F (2011). The nonspecific effects of vaccines and the expanded program on immunization. J Infect Dis.

[16] Shann F (2011). The non-specific effects of vaccines in low income countries. Arch Dis Child.

[17] Sankoh O, Welaga P, Debpuur C, Zandoh C, Gyaase S, Poma MA (2014). The non-specific effects of vaccines and other childhood interventions: the contribution of INDEPTH health and demographic surveillance systems. Int J Epidemiol.

[18] Benn CS, Aaby P (2007). Sex-differential responses to preventive health interventions. Maybe we treat boys and girls differently when we treat them equally?--secondary publication. Dan Med Bull.

[19] Benn CS, Martins C, Rodrigues A, Ravn H, Fisker AB, Christoffersen D (2009). The effect of vitamin a supplementation administered with missing vaccines during national immunization days in Guinea-Bissau. Int J Epidemiol.

[20] Dugas M, Dubé E, Kouyaté B, Sanou A, Bibeau G (2009). Portrait of a lengthy vaccination trajectory in Burkina Faso: from cultural acceptance of vaccines to actual immunization. BMC Int Health Hum Rights.

[21] Angwenyi V, Asante KP, Traoré A, Febir LG, Tawiah C, Kwarteng A (2015). Health providers' perceptions of clinical trials: lessons from Ghana, Kenya and Burkina Faso. PLoS One.

[22] Yaya Bocoum FI, Kouanda S, Hinson L, Collymore Y, Ba-Nguz A, Bingham A (2014). Community perceptions of malaria vaccines: qualitative research from the sanitary districts of Kaya and Hounde in Burkina Faso. Glob Health Promot.

[23] Ouédraogo LT, Ouédraogo SM, Ouédraogo ZT, Traore-Ouédraogo R, Kam L, Sawadogo A (2006). Factors for non-observance of the extended program timetable for vaccination in health districts: the case of Boussé in Burkina Faso. Med Mal Infect.

[24] Giles-Vernick T, Traoré A, Bainilago L (2016). Incertitude, hepatitis B, and infant vaccination in west and Central Africa. Med Anthropol Q.

[25] Sie A, Louis VR, Gbangou A, Muller O, Niamba L, Stieglbauer G, et al. The health and demographic surveillance system (HDSS) in Nouna, Burkina Faso, 1993-2007. Glob Health Action. 2010;3 10.3402/gha.v3i0.5284 PMC2940452 20847837

[26] Sié A, Niamba L, Diboulo E, Bagagnan C, Yé M (2010). Système de surveillance démographique et de santé(SSDS) de Nouna. Rapport annuel.

[27] Schoeps A, Ouedraogo N, Kagone M, Sie A, Muller O, Becher H (2013). Socio-demographic determinants of timely adherence to BCG, Penta3, measles, and complete vaccination schedule in Burkina Faso. Vaccine.

[28] Sié A, Niamba L, Yé M, Bagagnan C (2011). Système de surveillance démographique et de sante (SSDS) de Nouna CRSN. Rapport Annuel.

[29] Burton A, Monasch R, Lautenbach B, Gacic-Dobo M, Neill M, Karimov R (2009). WHO and UNICEF estimates of national infant immunization coverage: methods and processes. Bull World Health Organ.

[30] Ministère, de, la, Santé Annuaire statistique 2013. 2014. p. 337.

[31] Hoepfl MC (2007). Choosing qualitative research: a primer for technology education researchers. J Technol Educ.

[32] Pope C, Mays N (1995). Reaching the parts other methods cannot reach: an introduction to qualitative methods in health and health services research. BMJ.

[33] van Der Maren JM (1996). Les méthodes d'analyse exploratoire. Méthodes de recherche pour l'éducation.

[34] Bardin L (1983). L'analyse de contenu.

[35] van DMJ-M. Le codage et le traitement des données. Méthodes de recherche pour l'éducation: Presses de l'Université de Montréal, De Boeck Université, editors; 1996.

[36] Rosenstock IM, Glanz K, Lewis FM, Rimer BK (1990). The health belief model: explaining health behavior through expectancies. Health behavior and health education: theory, Research and Practice.

[37] Sya D. Stratégies et déterminants de la vaccination au Burkina Faso 1993 – 2003, Thèse présentée à la Faculté des études supérieures et postdoctorales en vue de l’obtention du grade de Philosophiae Doctor (Ph.D) en Santé Publique option Organisation des soins de santé, 2010: Université de Montréal; 2010.

[38] Mounier-Jack S, Burchett HE, Griffiths UK, Konate M, Diarra KS (2014). Meningococcal vaccine introduction in Mali through mass campaigns and its impact on the health system. Glob Health Action.

[39] Boa A (2006). Perceptions du Programme élargi de vaccinations (PEV) et de ses dysfonctionnements dans le district sanitaire de Bouna (Nord-Est de la Côte d’Ivoire). Bull Soc Pathol Exot.

[40] Tagbo BN, Uleanya ND, Omotowo IB (2013). Mothers’ knowledge and perception of adverse events following immunization in Enugu, south-east, Nigeria. Vaccines & Vaccination.

[41] Ouédraogo N, Kagoné M, Sié A, Becher H, Müller O (2013). Immunization coverage in young children: a study nested into a health and demographic surveillance system in Burkina Faso. J Trop Pediatr.

[42] Garcia LD, Velandia-Gonzalez M, Trumbo SP, Pedreira MC, Bravo-Alcantara P, Danovaro-Holliday MC (2014). Understanding the main barriers to immunization in Colombia to better tailor communication strategies. BMC Public Health.

[43] Sia D, Fournier P, Sondo BK (2011). Cultures locales de vaccination : le rôle central des agents de santé en milieu rural au Burkina Faso. Glob Health Promot.

[44] Léonard Fourn SH, Fournier P, Gansey R (2009). Determinants of parents' reticence toward vaccination in urban areas in Benin (West Africa). BMC Int Health HUm Rights.

[45] Robert J, Ledogar JF, Andersson N (2009). Knowledge synthesis of benefits and adverse effects of measles vaccination: the Lasbela balance sheet. BMC Int Health Hum Rights.

[46] Abdel Karim Koumaré DT, Haidara F, Sissoko F, Traoré I, Dramé S, Sangaré K, Diakité K, Coulibaly B, Togola B, Maïga A (2009). Evaluation of immunization coverage within the expanded program on immunization in Kita circle, Mali: a cross-sectional survey. BMC Int Health Hum Rights.

[47] Fisker AB, Hornshoj L, Rodrigues A, Balde I, Fernandes M, Benn CS, Aaby P. Effects of the introduction of new vaccines in Guinea-Bissau on vaccine coverage, vaccine timeliness, and child survival: an observational study. Lancet Global Health. 2014;2(8):e478–87. 10.1016/S2214-109X(14)70274-8 25103521

[48] WHO (2003). The quality of antimalarials. A study in selected african countries.

[49] Munthali CA, Mvula P. Knowledge, attitudes and perceptions study on, Immunisations and Diarrhoea, Centre for Social Research, Zomba,July 2012. 2012.

[50] Ministère de la Santé. Ministère de la Santé (2003). Direction de la Prévention par la Vaccination (DPV).

[51] Favin M, Steinglass R, Fields R, Banerjee K, Sawhney M (2012). Why children are not vaccinated: a review of the grey literature. International health.

[52] Fourn L, Haddad S, Fournier P, Gansey R. Determinants of parents' reticence toward vaccination in urban areas in Benin (West Africa). BMC Int Health Hum Rights. 2009;(Suppl 1):9, S14. 10.1186/1472-698X-9-S1-S14 PMC3226233 19828058

